# Mechanisms and impact of long COVID: pathophysiology, neuropsychiatric effects and vaccination

**DOI:** 10.3389/fimmu.2026.1710777

**Published:** 2026-06-19

**Authors:** Pretty Ponnachan, Anam Dhawlarker, Hadida Yasmin, Akash Shah, Aastha Malhotra, Abhishek Shastri, Basel K. Al-Ramadi, Uday Kishore

**Affiliations:** 1 Department of Veterinary Medicine (CAVM), United Arab Emirates University, Al Ain, United Arab Emirates; 2 Department of Medical Microbiology and Immunology, College of Medicine and Health Sciences, United Arab Emirates University, Al Ain, United Arab Emirates; 3 National Institute of Virology (NIV), Indian Council of Medical Research (ICMR), Pune, India; 4 Institute of Health Sciences, Presidency University, Kolkata, West Bengal, India; 5 Barts and The London School of Medicine and Dentistry, Queen Mary University of London, London, United Kingdom; 6 Ashford and St Peter’s NHS Foundation Trust, Ashford, United Kingdom; 7 Department of Zoology, Hans Raj College, University of Delhi, Delhi, India; 8 Central and North West London National Health Service (NHS) Foundation Trust, London, United Kingdom; 9 Zayed Centre for Health Sciences, United Arab Emirates University, Al Ain, United Arab Emirates; 10 ASPIRE Precision Medicine Research Institute Abu Dhabi, United Arab Emirates University, Al Ain, United Arab Emirates

**Keywords:** long COVID, SARS-CoV-2, complement system, neuropsychiatric effects, Epstein–Barr virus, autoimmunity, immune dysregulation

## Abstract

Long COVID or post-acute sequelae of COVID-19 is defined as an after-effect of acute COVID-19 infection. Its broad clinical symptoms include brain fog, shortness of breath, fatigue, joint, chest, or muscle pain, dysautonomia and neuropsychiatric symptoms such as anxiety, depression and post-traumatic stress disorder. It is estimated that 1 in every 5 COVID-19 survivors exhibit symptoms within the Long COVID bracket. An array of risk factors such as smoking habit, age, obesity, female sex, and prior hospitalization may increase the probability of a person developing Long COVID. While the underlying mechanisms of Long COVID remain elusive, we examine the various possible pathophysiologies involved in Long COVID. We take up impactful neuropsychiatric issues as another spectrum of Long COVID symptoms and the likely effect of various forms of COVID-19 vaccines. In this review, the focus will be on the main mechanisms associated with the development of long COVID, which include latent Epstein-Barr virus reactivation, molecular mimicry, virus persistence, autoantibodies, and mitochondrial dysfunction. Understanding these mechanisms shed light on the continued persistence of COVID-19 related symptoms long after the resolution of acute infection. For instance, the reactivation of Epstein-Barr virus in the immunocompromised context seen post-acute SARS-CoV-2 infection could lead to the symptoms commonly observed in Long COVID such as fatigue and brain fog. The Epstein-Barr virus could possibly disrupt mitochondrial function, explaining the fatigue commonly observed in Long COVID patients. Other factors such as continued presence of viral particles in specific areas such as the gut may result in continued inflammation, leading to manifestations such as fatigue, cognitive impairment and gastrointestinal dysfunction. Additionally, heightened and persistent presence of autoantibodies post-acute infection results in persistent symptoms and could potentially trigger the onset of autoimmune disorders. We also aim to revisit the diverse and prolonged effects of the COVID-19 pandemic that continue to affect the well-being and quality of human life.

## 1 Introduction

When SARS-CoV-2 first appeared in Wuhan, China, in December 2019, it rapidly spread worldwide, prompting the World Health Organization (WHO) to declare it a pandemic by March 2020 (1). While many patients experienced loss of life during the acute phase of the SARS-CoV-2 infection, a subset of patients, have reported continued experience of COVID-like symptoms well past the recovery stage. These patients are reported to experience a range of symptoms, from severe fatigue and brain fog to cardiovascular problems, turned to social media to coin the term “Long COVID (LC),” reflecting the challenges faced after both mild and severe SARS-CoV-2 infections. Although alternative terms such as PASC (post-acute sequelae of SARS-CoV-2 infection) have been proposed, “long COVID” has gained widespread recognition, largely due to its popularization on social media platforms. The WHO describes LC as a condition experienced by individuals with a history of confirmed or probable SARS-CoV-2 infection, usually 3 months from the disease onset, with symptoms lasting for at least 2 months and not explained by other diagnosis (2).

Meta-analysis data suggest that approximately 30-40% of the individuals who test positive for COVID-19 later develop LC, with a higher prevalence among hospitalized patients (approximately 44%) and lower in non-hospitalized patients (approximately 29%) (3–5). Another factor that influences whether a person develops LC is the vaccination status, with fully vaccinated individuals showing a 62% lower risk compared to the unvaccinated population (5). Other factors such as gender, ethnicity, immunosuppression and lower socioeconomic status may also contribute to LC (6, 7).

The WHO defines “post-COVID-19 condition” as unexplained symptoms lasting at least 2 months and present at least 3 months following SARS-CoV-2 infection (2). LC is characterized by a range of symptoms, including persistent fatigue, cognitive dysfunction, respiratory issues such as shortness of breath, and problems affecting the neurological, cardiovascular, and immune systems (8–10). Given the wide spectrum of reported symptoms and its heterogeneous nature, LC presents a significant challenge in terms of precise characterization and definition. This diversity makes it difficult for researchers and healthcare professionals to establish a unified clinical profile or diagnostic criteria for LC, highlighting the complexity of this post-viral syndrome. Research indicates that up to 80% of COVID-19 survivors may experience at least one long-term symptom (11). This persistence of disease symptoms can be indicative of the existing viral reservoirs leading to a dysregulated and compromised immune system.

Several studies indicate that SARS-CoV-2 could interfere with the typical operation of mitochondria, resulting in a wide range of LC symptoms, such as tiredness, cognitive impairment, memory problems, concentration difficulties, muscle pain, loss of smell, altered taste, and headaches; these symptoms are believed to be connected to inflammation in the nervous system and overall body caused by the virus, as well as possible increased blood clotting due to the virus. For example, research has identified abnormalities in mitochondrial respiration, bioenergetics, and mitochondrial-related gene expression in peripheral blood mononuclear cells (PBMCs) from LC patients (12–16). Unravelling the complexities of mitochondrial impairment in LC could pave the way for more precise and effective treatment strategies, as mitochondrial dysfunction has far-reaching consequences across multiple organ systems.

The underlying mechanisms of LC remain largely unclear. Multiple cohort studies have identified various risk factors associated with persistent COVID-19 symptoms, with female sex, obesity, and severe initial COVID-19 disease emerging as potentially significant contributors (17–19). Metabolic risk factors, such as insulin resistance and diabetes mellitus, have been linked to an increased likelihood of developing LC (20–23). Unsurprisingly, patients who were hospitalized during the acute phase of COVID-19 are considered at a higher risk of experiencing LC symptoms (22, 24). However, it is important to note that even patients with mild acute infections can experience lingering LC symptoms (25, 26). Other factors influencing LC development include different SARS-CoV-2 variants, vaccination status, and the number of vaccine doses received (7, 27, 28).

The constellation of LC symptomology targeting multiple organ systems clearly suggests a complex interplay of molecular processes, including viral persistence, immune dysregulation, and endothelial dysfunction (29). By exploring these facets, we aim to deepen our understanding of the underlying mechanisms involved in LC.

## 2 Factors influencing the susceptibility to developing LC

### 2.1 Involvement of renin-angiotensin system

SARS-CoV-2 is an enveloped virus composed of four structural proteins, envelope (E), membrane (M), nucleocapsid (N), and spike (S). The S protein consists of two subunits, S1 and S2, responsible for receptor binding and membrane fusion, respectively (30, 31). Infection begins when the receptor-binding domain (RBD) of the S1 subunit attaches to the human angiotensin-converting enzyme 2 (ACE2) receptor (30). ACE2 receptors are highly expressed in the heart, kidneys, and lungs, play a crucial role in the renin-angiotensin system (RAS) (32).

Within the RAS, ACE2 converts angiotensin I to angiotensin II, primarily in the lungs (33, 34). The effects of angiotensin II depend on whether it binds to angiotensin II type 1 receptor (AT1R) or Angiotensin II receptor type 2 receptor (AT2R); AT1R binding leads to increased oxidative stress and vasoconstriction, while AT2R binding counteracts these effects (33, 34). 

The S protein undergoes significant conformational changes during the infection process, with its RBD alternating between “open” and “closed” states (32). This dynamic structure allows the virus to effectively bind to ACE2 and initiate cell entry, facilitated by host proteases such as transmembrane serine protease 2 (TMPRSS2) (30, 35). TMPRSS2 mediated cleavage facilitates S protein activation. The SARS-CoV-2 is a clear example of tissue tropism, primarily targeting the respiratory epithelium, especially the type 2 alveolar cells, where ACE2 and TMPRSS2 are over-expressed, hence promoting cytopathic damage to the lungs (36, 37). Once inside the cell, the virus uses the cellular replication machinery to multiply and assemble viral particles, which then go on to infect other cells and aggravate the infection (38).

The RAS is critically involved in the severity of COVID-19 symptoms and associated pathologies (39). SARS-CoV-2 activates the RAS by binding via its S protein to the ACE2 receptor, which normally plays a role in degrading angiotensin II (Ang II). This binding impairs the ability of ACE2 to cleave Ang II, leading to its accumulation in the body (40). The increased Ang II then overstimulates the AT1R, resulting in a range of detrimental effects. Overactivation of AT1R can lead to vasoconstriction, hypertension, and cardiac hypertrophy, as well as lung fibrosis and tissue damage in the kidney and liver. Additionally, it can contribute to neurological issues such as ageusia (loss of taste) and anosmia (loss of smell), along with metabolic complications such as obesity and diabetes (39, 41–43). The overactivation of AT1R also has implications for skin health, potentially resulting in lesions.

The widespread expression of ACE2 across various tissues increases the likelihood of RAS dysregulation following SARS-CoV-2 infection, explaining the virus’s pleiotropic effects. This imbalance within the RAS may explain some of the long-term complications observed in patients following COVID-19, as the protective functions of ACE2 are compromised while the harmful effects of AT1R overstimulation persist (44).

The imbalance of the RAS in COVID-19, likely driven by virus-mediated downregulation of ACE2, may promote activation of the pro-inflammatory ACE/Ang II/AT1R axis, leading to the development of cytokine storm syndrome and subsequent cellular damage (45). This dysregulation results in overproduction of pro-inflammatory cytokines, including interleukin-1β (IL-1β), interleukin-6 (IL-6), interleukin-12 (IL-12), and interferon gamma (IFN-γ), which contribute to severe clinical outcomes of SARS-CoV-2 infection over time, such as pulmonary inflammation and significant lung damage due to fibrosis (46–48). Elevated levels of these pro-inflammatory cytokines in the plasma exacerbate systemic inflammation, potentially leading to acute respiratory distress syndrome (ARDS), multi-organ failure, and death (47, 49, 50).

Furthermore, serum ACE2 activity correlates with infection severity and mortality rates, indicating that impairment of the RAS may be more pronounced following severe SARS-CoV-2 infection (51).

### 2.2 Gender bias for LC

Gender-based differences in susceptibility to LC have been observed, with females showing a higher probability to develop the condition compared to males. However, during earlier outbreaks caused by other coronaviruses such as SARS and MERS, men experienced more severe symptoms and higher mortality rates (52). These dissimilar patterns suggest that gender may play an important role in the different phases and manifestations of the coronavirus infection in distinct ways. More women than men were infected by SARS in Hong Kong in 2003, but the death rate among men was 50% higher. While initial studies suggested that ACE2 expression, crucial for SARS-CoV-2 entry into cells, was higher in male lung samples, subsequent studies have shown mixed results across different tissues (53). The interplay between sex hormones, particularly oestrogen, and ACE2 expression adds complexity to this gender disparity. Notably, Elgendy et al. revealed that blocking oestrogen receptors in female mice increased mortality due to SARS-CoV infection, highlighting oestrogen’s potential protective role (54). Further investigation into these sex-based differences in expression is crucial for understanding their impact on COVID-19 susceptibility.

The relationship between ACE2 and TMPRSS2 expression and susceptibility to LC is not straightforward, as no significant gender differences were found in some tissues, such as salivary glands (55) and bronchial epithelium (56). This suggests that other factors, including behavioural differences such as smoking, may contribute to the observed gender disparity in LC cases (56).

The X chromosome plays a crucial role in understanding the differences in gender-based susceptibility of LC, with genes like ACE2 and Toll-like receptor 7 (TLR7) showing sex-specific expression patterns (57). Higher TLR7 expression in females, resulting from escape of X chromosome inactivation and consequent biallelic expression in immune cells, leads to increased production of interferon alpha (IFN-α) upon TLR7 activation (58). This enhanced type I interferon (IFN) response may facilitate more effective viral clearance in females but can also predispose them to overactive immune responses and persistent inflammation (50). Prolonged type 1 IFN signalling is associated with a higher risk of autoimmunity and may contribute to the development of LC symptoms in women (59, 60).

Epidemiological data from multiple countries highlighted stark sex-based differences in COVID-19 outcomes, with men being more likely to contract the virus than women (61). Evidence from China revealed significantly higher hospital admission and mortality rates among males (62, 63). For instance, in Italy, men accounted for 65% of deaths, with a male-to-female mortality ratio of 1.7 (64). A comprehensive review analysing case fatality rates across 38 countries found that men consistently showed a 1.7 times higher fatality rate than women from age 30 onwards (65).

Studies have shown that women over 50 experience more persistent symptoms like fatigue and shortness of breath, and women are approximately three times more likely to be diagnosed with LC compared to men (66).

The impact of LC on reproductive health remains largely unexplored, though research indicates that premenopausal women may face a higher risk of developing LC (67). Investigating the influence of gender roles on the persistence of symptoms following acute infection and LC could provide valuable insights into the distinct pathogenic mechanisms at play, ultimately contributing to more equitable health outcomes across genders.

#### 2.2.1 SARS-CoV-2 antigen can be transmitted vertically

Beyond these observations, cases of fetal transmission underscore the potential for mother-to-child transmission. Previous studies have shown vertical transmission rates of approximately 5.3% (68); however, recent meta-analysis data have revealed a lower transmission rate of approximately 4%, with considerable heterogeneity across studies. Maternal SARS-CoV-2 infection has also been associated with an increased risk of preterm birth and higher chances of caesarean delivery (69).

Lesieur et al. documented a fetal death at 24 weeks of gestation, occurring just seven days after the mother’s acute SARS-CoV-2 symptomatic infection (70). Pathological examination showed hepatocellular damage and hemosiderosis, with viral RNA present in the lung, liver, spleen, and trachea. Immunohistochemistry confirmed S protein presence in the stomach, liver, lymph node, and heart. Similarly, another report involved a neonate in the United States born at 25 weeks of gestation, who died four days after birth. The mother had asymptomatic COVID-19 and preeclampsia. Autopsy findings revealed severe alveolar damage and SARS-CoV-2 was localized in the neonate’s lungs, heart, and liver, indicating *in utero* transmission (71). The pathogenesis of severe COVID-19, including in pregnant women, involves the dysregulation of the Treg/Th17 cell ratio, favouring an increase in Th17 cells and resulting in uncontrolled systemic inflammation (72, 73). This Treg/Th17 imbalance might be associated with adverse pregnancy outcomes such as pregnancy loss, preterm birth, and preeclampsia (74, 75). An appropriate balance between these cell types is crucial for maintaining immune homeostasis and preventing excessive inflammation.

Just as there is a connection between acute COVID-19 and its immediate impact, studies have aimed to decipher the long-term effects of COVID-19. In a cohort of 409 pregnant women with acute COVID-19, 286 were followed for a mean of 92 weeks. Approximately 34.2% exhibited post-COVID-19 symptoms, with neurological (60%) and cutaneous (55%) manifestations being most common. Risk factors included migrant status, multiparity, severe symptoms, and lack of vaccination. Symptoms typically resolved over time, though some neurological and psycho-emotional symptoms persisted beyond 90 weeks. Perinatal outcomes showed no significant differences (76).

## 3 Pathophysiologies involved in LC

### 3.1 T cell activation and exhaustion during LC

As T cells contribute to viral clearance, persistent activation may trigger harmful effects, including the development of T-cell exhaustion (77). Studies have reported that people suffering from severe COVID-19 showed increased levels of exhausted T cells even six months after the initial infection. This exhaustion persists despite other markers of immune activation returning to normal levels (78). In another study, individuals with LC showed a 100% reduction in circulating levels of IFN-γ and IL-8 (79). IFN-γ, along with IL-12, helps drive the differentiation of Th1 cells, which in turn secrete IL-2 and TNF-α (80). Although there were no significant differences in the levels of IL-12 or TNF-α, plasma levels of IL-2 and IL-4 were found to be decreased in individuals with LC. This might be due to fewer T cells differentiating into Th2 cells, potentially leading to lower levels of Th2-associated cytokines (79).

#### 3.1.1 Comprehensive analysis of circulating and antigen-specific T cell subsets in COVID-19 and LC

Flow cytometry and multi-omics analysis revealed that LC individuals had a significantly higher proportion of CD4^+^ central memory T cells, T follicular helper (TFH), and regulatory T cells (Treg) cells than fully recovered individuals; however, no such variations were observed in SARS-CoV-2-specific CD4^+^ T cells. No significant differences in total SARS-CoV-2-specific CD4^+^ and CD8^+^ T cells among recovered and LC groups were found (81). Klein et al., 2023 showed an increase in the total CD4^+^ population as well as in exhausted CD4^+^ T cell. An increase in IL-4/IL-6-producing CD4^+^ T cells was also observed, however, no notable difference was observed in circulating CD45RA^–^CD127^–^CCR7^–^ effector memory subsets (82). SARS-CoV-2-specific CD8^+^ T cells in LC show signs of exhaustion, characterized by elevated PD-1 and CTLA-4 expression, potentially due to ongoing stimulation by viral antigens. CD4^+^ T cells expressed tissue-homing receptors CXCR4, CXCR5 and CCR6, indicating a potential for tissue migration. Unlike fully recovered individuals, LC patients also showed a loss of coordinated humoral and cellular immune responses, potentially suggesting a dysregulated IL-4 and IL-5 production by Th2 cells (81). Interestingly, LC subjects with highest SARS-CoV-2-specific PD-1^+^CTLA-4^+^CD8^+^ T cells had the lowest SARS-CoV-2-specific CD4^+^ Treg cells (81).

These scenarios may explain certain LC complications such as prolonged respiratory symptoms, chronic fatigue, difficulties in oxygen transfer and persistent inflammation, suggesting immune dysregulation as the driver of LC (83, 84).

#### 3.1.2 Prolonged antigen presentation leading to T cell exhaustion

**Figure 1:**
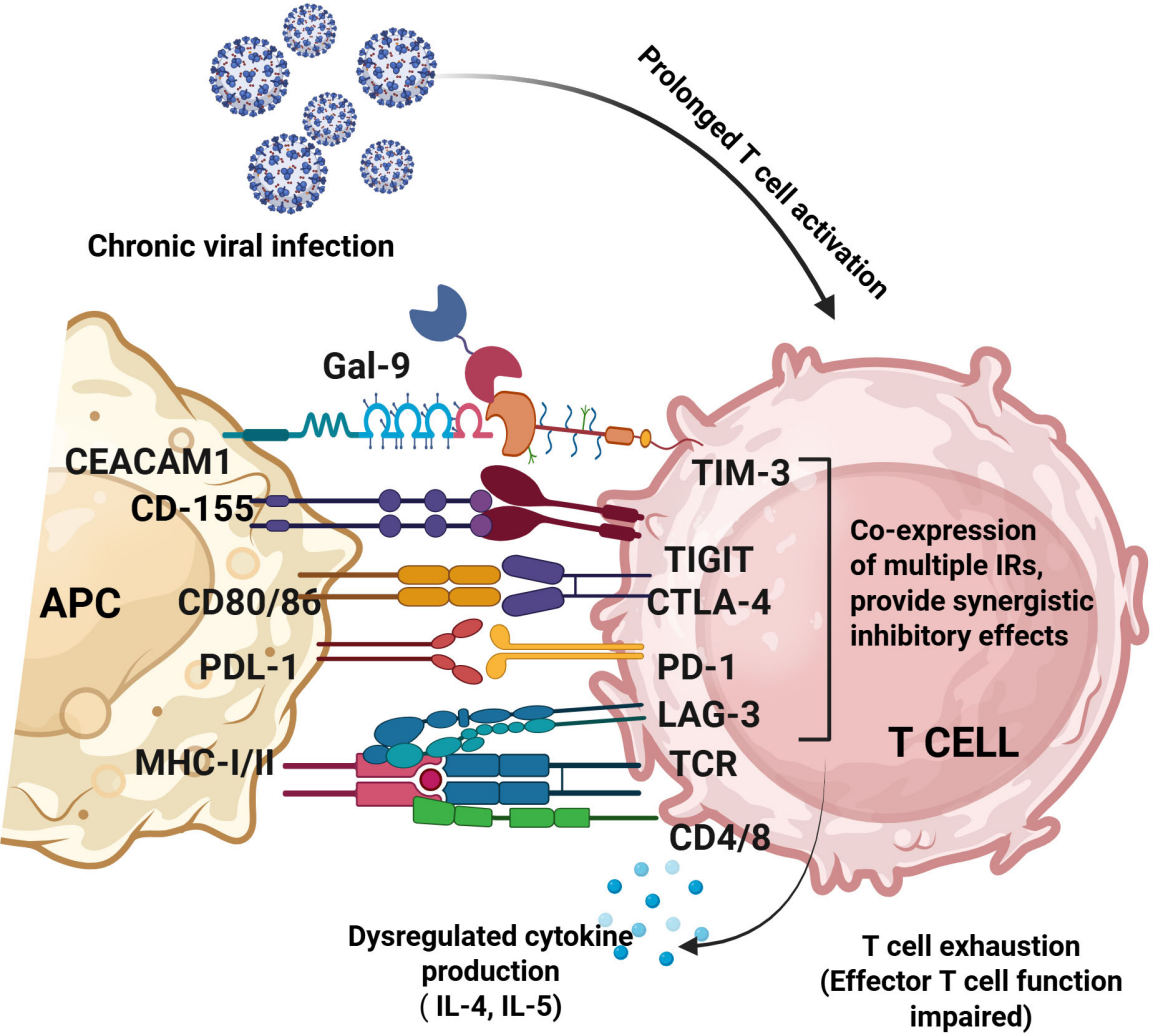
Prolonged antigen presentation leading to T cell exhaustion. Longer period of antigenic exposure during chronic viral infection keeps the T cells activated for a longer duration causes upregulation of inhibitory receptors (IRs) such as TIM-3 (T cell immunoglobulin domain and mucin domain-3), TIGIT (T-cell immune receptor with Ig and ITIM domains), CTLA-4 (Cytotoxic T-Lymphocyte-Associated Protein 4), PD-1 (programmed cell death protein1) and LAG-3 (lymphocyte activation gene-3). Expression of these exhaustion markers leads to loss of coordinated humoral and cellular immune responses (eg. dysregulated IL-4 and IL-5 production by Th2 cells). Eventually causing T cell exhaustion (impaired T cell effector functions). *Created in* *https://BioRender.com*.

A prolonged period of T cell activation is followed by increased expression of the T cell exhaustion markers such as PD-1, TIGIT, sTim-3, CTLA-4 and LAG-3 which typically appear when T cells are subjected to prolonged antigen stimulation (**Figure 1**) (81, 85, 86). Elevated levels of sTIM-3 were observed in a cohort systematically followed for 8 months after SARS-CoV-2 infection (86). In a subsequent study by the same research group, increased TIM-3 expression was noted on both CD4^+^ and CD8^+^ T cells in LC patients at 3 month post-infection. This elevation in TIM-3 also persisted in CD4^+^ T cells at 8 months but normalized by 24 months (87, 88). While the study found increased frequencies of S and N specific CD4^+^ T cells in LC, the reported changes in exhaustion markers such as TIM-3 refer to the overall CD4^+^ T cell population.

### 3.2 Complement dysregulation and its role in LC pathogenesis

Quantification of complement proteins, including markers of activation and regulation, in plasma samples from patients with a confirmed history of SARS-CoV-2 infection revealed that markers of classical (C1s-C1INH complex), alternative (Ba, iC3b), and terminal pathway [C5a, Terminal Complement Complex (TCC)] activation are significantly elevated in patients with LC (89). A similar study wherein 113 COVID-19 patients were followed up for up to 1 year post-acute SARS-CoV-2 infection, identified biomarkers associated with LC (90). At the 6-month follow-up, 40 patients who presented with LC symptoms had a dysregulated immune system characterized by persistent complement activation reflecting on an imbalanced formation of the TCC, with increased levels of soluble C5b-9 complexes and decreased C7-containing TCC formations which are capable of integrating into the cell membranes and inducing cell activation or lysis (90). Additionally, complement-mediated tissue injury in LC has been associated with elevated markers of tissue injury, such as von Willebrand factor and low antithrombin III levels, supporting the existence of a thrombo-inflammatory signature. This suggests that the TCCs are more prone to inserting into host cell membranes (91), potentially causing tissue damage and contribute to the symptoms experienced post SARS-CoV-2 infection.

#### 3.2.1 Neutrophil extracellular traps and complement activation

Recent research underscores the critical role of neutrophil extracellular traps (NETs) in complement dysregulation, particularly in the context of COVID-19-related immuno-thrombosis (92, 93). NETs, web-like structures released by neutrophils, can exacerbate complement activation (94). NETs play an important role in COVID-19 immuno-thrombosis by exacerbating complement activation and promoting coagulation (93, 95). Complement by-products produced by the activated complement pathway, have important immunostimulatory roles in vascular permeability and inflammatory cell recruitment (96, 97). Complement fragments like C3a and C5a, can act as immunostimulatory mediators that can increase vascular permeability, recruit inflammatory cells and stimulate neutrophils to release NETs (93, 95). Elevated plasma levels of C3a and C5a, along with NET markers, have been observed in COVID-19 patients and are linked with an increased risk of thrombotic complications and disease severity (95, 98). Additionally, dysregulation of the complement system can lead to autoantibody formation as NETs can expose self-antigens that are usually sequestered from the immune system, potentially triggering the production of autoantibodies and resulting in chronic inflammation and further immune dysfunction. The generation of NETs in COVID-19 patients is promoted by anaphylatoxins C3a and C5a. These NETs are linked to coagulation disorders and multiple organ dysfunction (98, 99). This interaction highlights how complement components, such as C3a and C5a, act as crucial mediators that connect inflammation and blood clotting in COVID-19, contributing to the development of cytokine storm as well as coagulopathy that are central features of the disease (97).

The presence of antibodies against herpesviruses, specifically cytomegalovirus (CMV) and Epstein-Barr virus (EBV), as well as increased autoantibody prevalence, can drive continuous inflammatory processes by forming immune complexes that activate the complement system, potentially contributing to persistent immune system dysregulation in LC (90). This interplay between NETs, autoantibody and the complement form a self-amplifying loop where NETs enhance complement activation, and complement components further stimulate NET formation. This bidirectional interaction perpetuates a cycle of inflammation and immune activation, contributing to ßpersistent tissue damage and disease progression (100).

#### 3.2.2 Complement regulation as a target for disease monitoring and therapies

**Figure 2:**
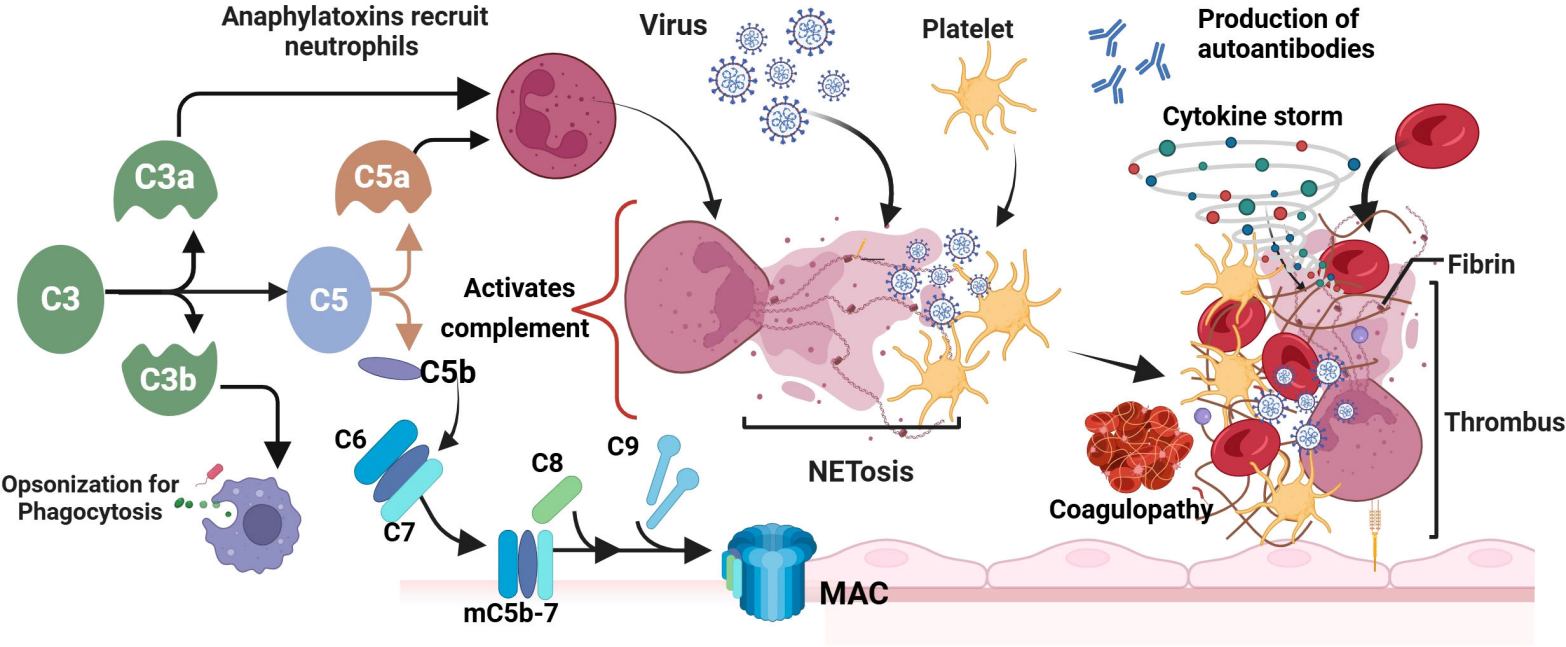
Neutrophil extracellular traps (NETs) and Complement activation. Complement fragments like C3a and C5a, can act as immunostimulatory mediators that can increase vascular permeability, recruit inflammatory cells and stimulate neutrophils to release NETs (web-like structures released by neutrophils) exposing self-antigens and attracting platelets. The generation of NETs in COVID-19 patients promoted by anaphylatoxins C3a and C5a, contribute to the development of both the cytokine storm and coagulopathy that are central features of the COVID-19. The presence of antibodies against residual viral antigens or autoantigens can lead to a continuous inflammatory state, potentially causing immune system dysfunction. This interplay between NETs, autoantibody generation and the complement system form a self-amplifying loop where NETs enhance complement activation, and in turn, complement components further stimulate NET formation. Created in https://BioRender.com.

As the complement system is crucial for defending the host against invading pathogens, its excessive activation can lead to detrimental effects, its excessive stimulation during SARS-CoV-2 infection can lead to prolonged systemic inflammation (**Figure 2**). Importantly, inhibitors such as recombinant carboxypeptidase B have been shown to mitigate vascular cell damage by reducing C3a- and C5a-induced NET formation (99). Supplements such as Vitamin C can neutralize the oxidative damage, decreasing the ability to induce NETosis (101). In addition, investigating combination therapies that integrate multiple strategies could enhance overall effectiveness in managing the symptoms.

### 3.3 Herpesvirus reactivation and its role in LC immune dysregulation

The reactivation of latent herpesviruses has been associated with sustained immune dysregulation for long periods of COVID-19 (102). The reactivation process refers to the mechanism by which the latent viruses [(e.g. EBV and Varicella Zoster Virus (VZV)], which remain dormant in B lymphocytes and epithelial cells following initial infection, become active and enter their replication cycle. The reactivation of these dormant viruses can be triggered by concurrent infections, physical or psychological stress or immunosuppression; the reactivation can be detected by the presence of elevated EBV DNA levels in the blood.

For instance, Myalgic Encephalomyelitis/Chronic Fatigue Syndrome (ME/CFS) has been linked to both LC and other viral and bacterial infections (103). Furthermore, dysautonomia, a condition observed in several post-viral infections, has also been reported in LC patients (104).

EBV and VZV, affect more than 90-95% of the global population, causing symptoms similar to ME/CFS. Post primary infection, these viruses can remain latent within the host and can be reactivated by a stressor such as another serious viral infection (e.g. SARS-CoV-2), leading to chronic inflammation and neurological disorders (103).

EBV infects B lymphocytes and epithelial cells, potentially triggering autoimmune disorders such as multiple sclerosis, Systemic Lupus Erythematosus (SLE), and rheumatoid arthritis (105). Psychological stress such as duress experienced during extended hospitalization under a critical health crisis is known to trigger EBV reactivation (106). Several studies have indicated a coherent association between EBV reactivation and severe SARS-CoV-2 infection (107). EBV reactivation was seen in patients with both mild and severe SARS-CoV-2 infection; however, reactivation was seen to be more pronounced in individuals with severe infection compared to the milder cases (108). Immunocompromised individuals such as transplant recipients were more susceptible to aggressive virus reactivation (109).

Association between EBV reactivation and SARS-CoV-2 infection has been demonstrated by several studies. Palucci et al. reported that 95.2% of intensive care unit (ICU) patients with COVID-19 tested positive for EBV DNA. These patients had evidence of immune dysregulation, including reduced CD8^+^ T cells and NK cells (110). These findings indicate a possible association between EBV reactivation and dysregulated immune response observed during COVID-19.

EBV reactivation was evaluated by measuring serological markers such as viral capsid antigen (VCA) IgM and early antigen-diffuse (EA-D) IgG, which are indicative of early lytic phase activation. EA-D IgG, represents heightened viral replication and is often detected during reactivation episodes (111). However, the detection of serological markers alone may not be enough to definitively indicate active EBV infection. Zubechenko et al. showed herpesvirus reactivation using quantitative PCR detection of viral DNA in peripheral blood, oropharyngeal mucosa and saliva, providing a stronger evidence towards active viral replication (112). Similarly, among 34 patients admitted to the ICU with severe COVID-19, EBV DNA was detected at least once in 28 patients (82.4%), supporting the occurrence of EBV reactivation (107).

Certain epitopes in the S1 and S2 subunits of the SARS-CoV-2 S protein are recognized by antibodies that also bind to antigens from viruses such as EBV, CMV, and HSV-1, demonstrating antibody cross-reactivity rather than mere sequence similarity (113). Such cross-reactivity between SARS-CoV-2 S protein epitopes and other viruses such as EBV could inadvertently lead to latent virus reactivation, and hence, immune dysregulation.

**Figure 3:**
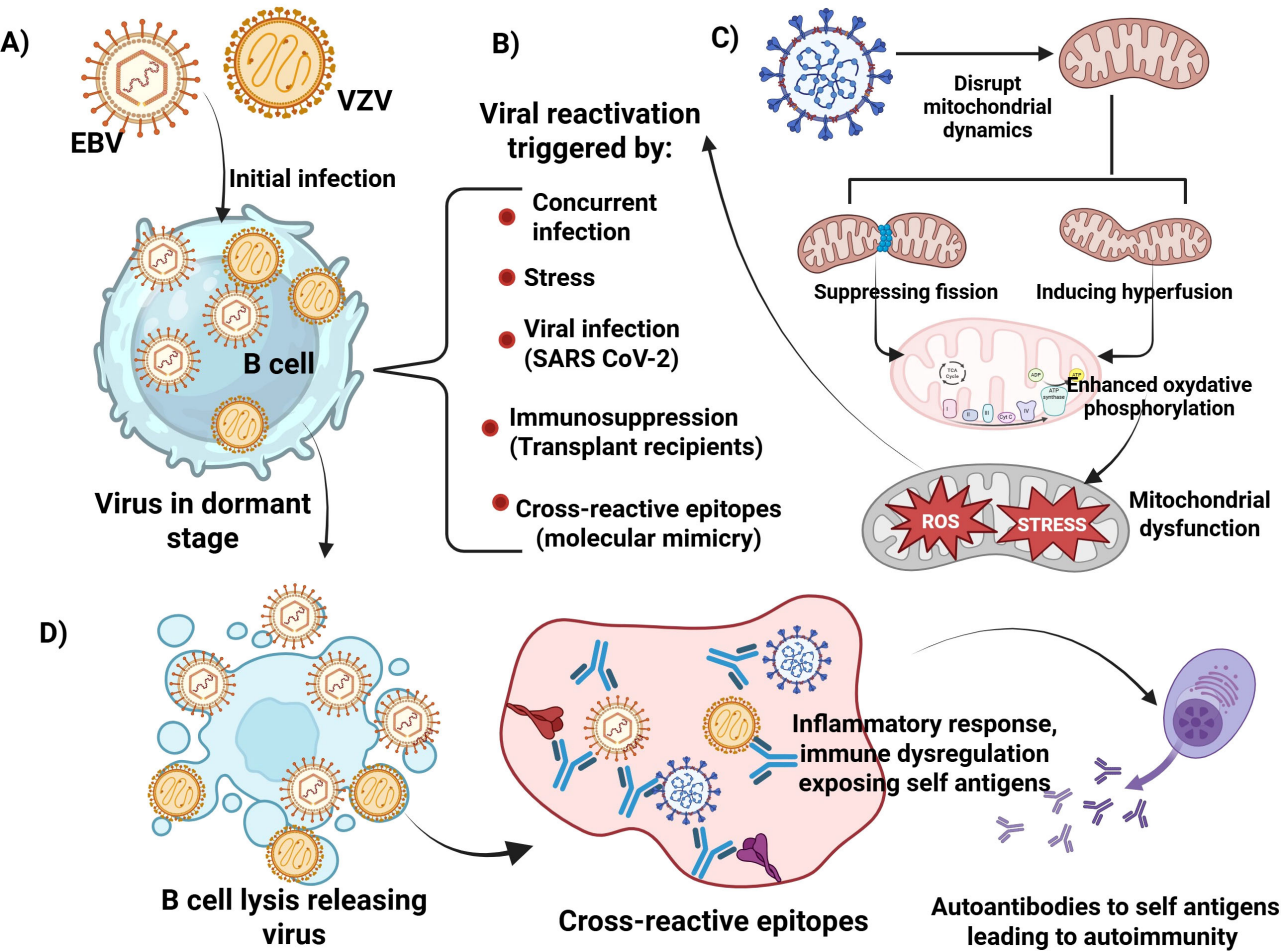
Viral reactivation and autoimmunity. **(A)** Following initial infection, the viruses remain dormant in B lymphocytes and epithelial cells. These latent viruses (e.g. Epstein Bar Virus (EBV) and Varicella Zoster Virus (VZV) can be reactivated through several triggers. **(B)** The reactivation of these dormant viruses can be triggered by concurrent infections, physical or psychological stress and SARS-CoV-2 infection. Immunocompromised individuals such as transplant recipients were privy to aggressive virus reactivation. Certain epitopes of S1 and S2 subunits of SARS-CoV-2 S proteins recognized by antibodies binding to antigens of viruses (EBV, CMV and herpes simplex virus-1 (HSV-1), demonstrating antibody cross-reactivity can inadvertently lead to latent virus reactivation. **(C)** SARS-CoV-2 infection can disrupt mitochondrial dynamics by either suppressing fission or by inducing mitochondrial hyperfusion. This affects the functioning of the electron transport chain, producing reactive oxygen species (ROS) and leading to oxidative stress. This mitochondrial dysfunction can also be a trigger for viral reactivation. **(D)** The reactivation of latent herpes viruses has been associated with sustained immune dysregulation for long periods of COVID-19. Once reactivated the viruses enter their replication cycle and are eventually released with the lysis of B cells, thus, exposing the viral antigens once again. These post-acute viral infections may trigger the onset of autoimmunity due to production of autoantibodies targeting host tissues after contracting COVID-19. The cross-reactivity between pathogen and host-antigens could be a possible explanation for the persistent inflammation observed in LC. Created in https://BioRender.com.

A study to compare EBV reactivation rates in patients suffering from LC versus a control group of COVID-19 survivors without LC symptoms, divided patients into two groups, one describing long-term study (3 months or more post-COVID diagnosis) and the other a short-term study (3 weeks to 3 months post-COVID diagnosis) (114). EBV reactivation was assessed by detecting positive EBV EA-D IgG or EBV VCA IgM titres. The long-term study showed that 66.7% of LC subjects showed EBV reactivation versus 10% of control subjects. The short-term study showed a similar pattern with 66.7% of LC showing positive EBV reactivation versus 11.1% of control subjects. Both studies support the that viral reactivation can occur soon after or simultaneously with SARS-CoV-2 infection (114), and reaffirm that EBV reactivation is a possible mechanism for the progress of LC symptomology. EBV reactivation can also lead to mitochondrial fragmentation, affecting the energy metabolism, leading to severe fatigue and brain fog (**Figure 3**) (115).

There are other herpesviruses that have also been implicated in viral reactivation, such as VZV. For instance, 3 patients with SARS-CoV-2 infection and vesicular eruption revealed VZV and other herpesvirus DNA in vesicle contents (116). Interestingly, dermatological manifestations such as Pityriasis Rosea-Like Lesions (PR-LE) have been reported following SARS-CoV-2 immunization. In a cohort study of 405 COVID-19 patients, PR-LE was observed in 4.9% of individuals, with viral reactivation of VZV and herpes simplex virus (HSV) documented in 10.1% and 3.7% of the cohort, respectively (117). In addition, there were concerns about the reactivation of VZV and HSV following vaccination with Tozinameran (Pfizer-BioNTech COVID-19 vaccine). However, Brosh-Nissimov et al., 2021, found no significant increase in oropharyngeal shedding or reactivation of these herpesviruses in immunocompetent adults one week after vaccination compared to pre-vaccination samples (118). VZV infection is comparatively much less reported in LC as compared to EBV reactivation, although it might have significant effects on the central nervous system (CNS).

### 3.4 Development of post-viral autoimmunity

Autoimmunity and viral infections are closely intertwined. Autoimmune conditions can exacerbate the severity of acute SARS-CoV-2 infection, while post-acute viral infections may trigger the onset of autoimmune disorders, as observed with other viral pathogens.

Acute viral infections such as Parvovirus B19, EBV, CMV, Human herpesvirus-6 (HHV-6), Hepatitis A and C virus, and Rubella virus have been reported in initiating chronic inflammatory or autoimmune diseases such as rheumatoid arthritis, systemic lupus erythematosus (SLE), Sjögren’s syndrome, multiple sclerosis (MS), Hashimoto thyroiditis and autoimmune hepatitis (119–122).

A large-scale, binational, longitudinal, propensity-matched cohort study using nationwide health insurance claim databases from South Korea (over 10 million patients) and Japan (over 12 million patients) found that individuals with prior COVID-19 infection had a 25% to 30% increased risk of developing new-onset autoimmune inflammatory rheumatic diseases (AIRDs) within the first year post-COVID-19 infection (123, 124). These findings underscore the role of COVID-19 as a catalyst for autoimmune disease development (124).

Multisystem inflammatory syndrome in children (MIS-C), characterized by high fever and tissue inflammation with a suspected autoimmune basis, has been increasingly linked to paediatric LC cases (125). The typical onset of MIS-C occurs 2 to 6 weeks post-infection, paralleling the emergence of LC symptoms in children. This temporal overlap, coupled with the shared involvement of dysregulated immune responses, suggests that MIS-C may represent a severe manifestation of paediatric LC or a related immune-mediated episode (126, 127).

Developing an autoimmune disorder post-acute viral infections may be considered a characteristic trait of LC. Several mechanisms may contribute to the onset of these autoimmune conditions, some of which are discussed below.

#### 3.4.1 Role of molecular mimicry of viral antigens in LC

Molecular mimicry is a key mechanism driving the development of pathogen-triggered autoimmune diseases. It functions by exploiting existing similarities between foreign or environmental antigens and host proteins, triggering an immune response that mistakenly targets self cells or tissues (128).

Molecular mimicry operates through several mechanisms, but the one most closely linked to the development of autoimmune diseases hinges on structural similarities between antigens and host components (129). These similarities may exist in amino acid sequences, epitopes, or glycosylation patterns (130). As the immune system targets the invading pathogens, it may attack host cells or tissues that resemble these pathogenic features triggering autoimmune processes.

Pre-existing exposure to common human coronaviruses (e.g., HCoV-OC43, HCoV-HKU1) and other viruses (e.g., influenza, EBV) can prime cross-reactive antibody responses to conserved epitopes shared between SARS-CoV-2 and previously encountered pathogens (131). For example, COVID-19 infection has been shown to modulate antibody responses to unrelated viruses like respiratory syncytial virus (RSV), CMV, and HSV-1, possibly due to molecular mimicry between SARS-CoV-2 proteins and antigens from these viral pathogens (132). This cross-reactivity between pathogen and host antigens could explain the persistent inflammation observed in LC.

Some people may have pre-existing cross-reactive immune response to SARS-CoV-2 prior to the pandemic. This phenomenon could be explained by molecular mimicry, whereby structural similarities between SARS-CoV-2 antigens and previously encountered pathogens generate cross-reactive antibodies and T cells (133). Pre-existing IgG antibodies specific to SARS-CoV-2 S protein were detectable in COVID-19 naïve patients, indicating a heterogeneous cross-reactive immune responses shaped by previous encounters with other related pathogens (113). This phenomenon of pre-existing immunity can be explained by considering exposure to other coronaviruses such as those causing the common cold coronaviruses such as HCoV-OC43 and HCoV-HKU1, or other viruses such as CMV, HSV-1, and EBV (113). Interestingly, SARS-CoV-2 epitopes in S1 and S2 regions could activate CD8^+^ T cells of healthy pre-pandemic people, suggesting that these people had prior exposure to related viruses or vaccination (134).

Further evidence for the role of molecular mimicry was provided by a study that identified 63 human proteins that shared similarities with portions of the SARS-CoV-2 S protein, which were identified in pre- and post-pandemic individuals. The identified proteins play key roles in the pathogenesis of Parkinson’s disease, Irritable bowel syndrome (IBS), chronic pulmonary disease, cancer and periodontitis (113).

A computational study of the SARS-CoV-2 S protein revealed potential molecular mimicry hotspots with a tentative high auto-antigenic potential (135). A TQLPP motif in the S protein has similar antibody-binding site as thrombopoietin, potentially contributing to the low platelet counts seen in COVID-19 (135). The ELDKY motif, shared between SARS-CoV-2 S and human proteins like PRKG1 (platelet regulation) and tropomyosin (cardiac function), could drive autoimmune responses contributing to LC-associated blood clotting and heart complications. These effects typically manifest months post-infection due to delayed antibody cross-reactivity (135).

Recent proteomic studies have identified potential biomarkers and pathways associated with LC, including evidence of hypoxia-driven vascular remodelling and inflammation (136). While the exact mechanisms linking molecular mimicry to LC remain to be established, this conceptual framework provides a basis for further investigating the complexity of the post-acute sequelae of SARS-CoV-2 infection.

#### 3.4.2 Molecular mimicry and immune cross-reactivity observed in LC

Autoantibodies have been implicated in the development of a variety of autoimmune disorders, for example Type 1 diabetes (T1D) is associated with autoantibodies against glutamic acid decarboxylase (GAD), islet cell antigens, and insulin (137), rheumatoid arthritis (RA) with rheumatoid factor (RF) and anti-citrullinated protein antibodies (ACPA) (138), MS with antibodies to myelin basic proteins (139), SLE with antinuclear antibodies (ANA), anti-dsDNA and anti-Smith antibodies (140), Graves’ disease (GD) with thyroid-stimulating IgG antibodies (141), psoriasis with autoantibodies against LL-37 and A disintegrin and metalloproteinase with thrombospondin motifs-like 5 (ADAMTSL5) (142), inflammatory bowel disease (IBD) with ANA and other autoantibodies (143), Sjögren’s syndrome (SS) with anti-Ro/SSA and anti-La/SSB (144) and celiac disease (CD) with anti-tissue transglutaminase (tTG) and anti-deamidated gliadin peptide peptide (DGP) antibodies (145).

In the context of SARS-CoV-2, the emergence of autoimmune disorders post-infection can be attributed to the production of autoantibodies targeting host tissues after contracting COVID-19. A key example of this phenomenon is the detection of anti-ACE2 antibodies in patients with LC (146). These antibodies are linked to common LC symptoms such as fatigue and myelitis (147), and may disrupt the normal functioning of the RAS, potentially amplifying late thrombo-inflammatory pathways (148).

Emerging research has identified the presence of SARS-CoV-2-specific immunoglobulin profiles in individuals with LC, where lower IgM and IgG3 antibody titres are associated with an increased risk of experiencing the long-term effects of COVID-19 (149). The decreased expression of type I IFNs, essential mediators of the antiviral immune response, has been correlated with the emergence of autoantibodies that recognize and interact with these IFNs, particularly IFN-α2 and IFN-ω (149, 150). These autoantibodies can compromise the body’s capacity to mount an effective immune response against the virus, resulting in viral persistence and various autoimmune-like conditions, including Guillain–Barré syndrome (151, 152), thrombocytopenia (153), and SLE.

Autoantibodies targeting type-I IFNs have been strongly linked to an increased likelihood of developing severe COVID-19 illness (150, 154–156). Type-I IFNs are essential signalling molecules that initiate and coordinate the host’s immune response against viral infections. Notably, individuals with pre-existing autoimmune disorders, such as SLE, have a higher prevalence of anti-IFN-α autoantibodies when co-infected with SARS-CoV-2 (154, 156). While anti-IFN autoantibodies may increase the risk of developing LC by increasing the severity of the initial COVID-19 illness, their direct relationship with the persistence of symptoms in LC is unclear. It is possible that the prolonged presence of these autoantibodies could delay viral clearance and promote viral persistence, ultimately contributing to the development of LC. Peluso and colleagues have shown that although anti-IFN antibodies are linked to severe COVID-19, their prevalence is relatively low among individuals with LC (157) (**Table 1**).

**Table 1:** Table comprising of key autoantibodies detected in COVID-19 and LC.

SI No.	Autoantibody	Molecular target	Related symptoms in LC	Mechanism of action	Reference
1.	Anti-nuclear antibodies (ANA)	Nuclear antigens (DNA, histones, extractable nuclear antigens)	Fatigue, joint pain, cognitive impairment, onset of autoimmunity	ANA bind to nuclear antigens released from dying cells; immune complexes can deposit in tissues, thus activating complement and inflammation.	(158–160)
2.	Anti-cytokine antibodies (ACA)	Type I IFNs (IFN-α/ω), IL-1β, IL-6, GM-CSF	Recurrent infections, fatigue, brain fog	Neutralizes cytokines, impairs antiviral defence, causing immune dysregulation.	(158, 161, 162)
3.	Anti-ACE2 antibodies	Angiotensin-converting enzyme 2 (ACE2)	Cardiovascular dysfunction, Severity of COVID-19, respiratory symptoms (ARDS)	Anti-ACE2 bind to ACE2 on endothelial cells, activate the classical pathway, inducing cell injury and inflammation	(163)
4.	Anti-phospholipid antibodies (APLA)	Cardiolipin, β2-glycoprotein I, prothrombin, annexin-V	Thrombosis, micro-clots, cardiovascular events	APLA binds to phospholipids and phospholipid-binding proteins and non-classical antigens on platelets and endothelial cells, triggering coagulation cascade, hypercoagulability.	(164–169)
5.	Rheumatoid factors (RFs)	Fc region of IgG	Joint pain, fatigue, increased risk of severe disease	RFs bind to IgG, immune complex mediated classical pathway activation and form immune complexes cause infiltration, inflammation and tissue damage.	(158, 170, 171)
6.	Anti-GPCR autoantibodies	G-protein coupled receptors (β2-AR, α1-AR, AT1R, M2R, MASR, ETA, NOC)	Fatigue, autonomic dysfunction, POTS, neurological/cardiac symptoms	These autoantibodies act as agonists/antagonists of GPCRs, causing chronic activation or inhibition of signalling, disrupting autonomic, cardiovascular, and neurological regulation.	(158, 172)
7.	Anti-SNURF IgG	SNRPN upstream reading frame protein	Neuropsychiatric symptoms (anxiety, brain fatigue, impaired concentration, depressed mood, impaired memory)	Anti-SNURF IgG autoantibodies may enter the central nervous system, bind to neural antigens, and induce neuroinflammation and dysfunction.	(158, 173)
8.	Anti-AGTR1 autoantibodies	Angiotensin II type 1 receptor (AT1R)	Fever, muscle aches, loss of smell or taste during COVID-19, post-infection, memory problems, headaches, fatigue, dizziness, and other neurological symptoms.	Anti-AGTR1 autoantibodies can attach to and activate AT1 receptor, increasing inflammation and damage to blood vessel lining, and thus making blood vessels stiff and leaky.	(174, 175)

Autoantibodies along with their molecular targets, associated symptoms and mechanism of action. The mechanisms describe how these circulating autoantibodies disrupt immune homeostasis by forming immune complexes or interfering with cell signalling. The table aids in understanding the persistent symptoms seen in LC.

Patients with mild to severe acute infection who later developed LC exhibited high levels of antibodies to G protein-coupled receptor (GPCRs) (126, 176, 177). This could inadvertently affect the catecholamine and acetylcholine signalling, resulting in dysfunction of the nervous system. Interestingly, a key feature of GPCR is chemo-sensing, and epithelial cells like tuft cells possess this unique chemo-sensing ability (178). Persistent infection affecting the gut could derail the homeostatic immune system and negate this chemo-sensing property of GPCR (179). This association of GPCR with chemo-sensing tuft cells in the intestine could explain taste and smell abnormalities experienced during LC (179).

A case study showed how DNA aptamers have the potential to neutralize these autoantibodies, suggesting a potential treatment option for LC by restoring regular immune function. Notably, autoantibodies targeting receptors such as CXCR3, AGTR1, β2-adrenoceptor, and muscarinic receptors were detected in LC patients (172, 176, 177).

A major limitation in establishing the relationship between SARS-CoV-2 infection and autoimmunity is the lack of baseline patient data prior to the pandemic, which complicates the assessment of latent autoimmunity post-infection. Some studies suggest that pre-existing autoantibodies may contribute to the development of LC, however, the clinical relevance of these autoantibodies remains unclear (180). While evidence indicates a role for autoantibodies in driving autoimmune disorders linked to LC, conflicting findings exist. For example, a multidimensional immune profiling study of 98 LC patients using rapid extracellular antigen profiling (REAP) did not identify stereotypical autoantibodies that differentiate LC patients from healthy controls (82).

#### 3.4.3 Viral reservoirs and persistence in LC:

Partial or full viral persistence has been documented following infections in the case of Polio, Chikungunya, Ross River, Measles, and Ebola. This persistence has been associated with prolonged or delayed health complications (181). For instance, five years after the Ebola virus outbreak ended in 2016, the virus re-emerged in individuals who had previously recovered from Ebola virus disease (182). Viral RNA has been detected in immune-privileged sites, including cerebrospinal and ocular fluids, months after recovery, leading to conditions like meningitis and uveitis. Similarly, West Nile virus has been found to persist in the CNS and kidneys, contributing to chronic symptoms such as cognitive impairments and kidney disease (181, 183).

In the context of LC, the relapsing and remitting nature of symptoms have led to the notion that viral persistence may serve as a key mechanism underlying its pathogenesis (184). This suggests that residual viral components in specific tissues could drive immune dysregulation and contribute to the chronic symptoms due to inflammation commonly experienced by survivors. While the debilitating and pronounced symptoms of acute COVID-19 infection persist, a suitable explanation would be the continued presence of the virus in various tissues. Studies have validated the presence of virus reservoirs by analysing biopsy and autopsy-derived tissue samples using PCR, tissue culture, or immunostaining. Stool and blood sample analysis using qPCR and antigen-antibody-based techniques also furthered this hypothesis of the existence of a potential viral reservoirs post-SARS-CoV-2 infection (185).

Among the symptoms described in LC, gastrointestinal diseases have been widely reported. Nearly 10% of IBD patients had previously contracted COVID-19 infection, and 40% of these patients later developed LC symptoms (186). This suggests the gut could represent a site for viral persistence. Viral proteins such as N and S proteins, have been detected in specific intestinal sites (187). A study involving a cohort of patients with IBD, viral RNA and proteins like N and S were detected in the intestinal mucosa up to 7 months after acute SARS-CoV-2 infection, although no live virus could be detected. This antigen persistence was associated with LC symptoms; individuals without detectable gut viral antigens did not report LC-like symptoms. Thus, study indicates that incomplete clearance of the viral antigens in the gut could aggravate or contribute to the development of LC symptoms (82, 187).

Interestingly, ACE2 receptors are present on enterocytes and hepatocytes (188), which could explain the onset of gastrointestinal problems like IBD and abnormal liver function. In support of viral persistence in the gut, studies have shown that 4 months post-acute infection, intestinal biopsies detected SARS-CoV-2 N protein, even in patients who reported mild infection (189). Similarly, viral particles have been detected in fecal matter of LC patients. SIMOA (single molecular array) was used in a research study to detect SARS-CoV-2 antigens in serum from 37 COVID-19 patients and 26 recovered controls (190).

Key evidence supporting the idea of a gut viral reservoir comes from B cell repertoire analysis and the direct detection of SARS-CoV-2 in gut biopsies. Ongoing antigenic stimulation, as indicated by stable memory B cell frequencies and increased somatic hypermutation, suggests that the virus may persist in the gastrointestinal tract (189). The precise mechanisms by which a potential gastrointestinal viral reservoir may contribute to the development of long-term complications following SARS-CoV-2 infection, such as LC, continue to be an active area of research. Further investigation is required to fully understand this complex relationship (191).

A year, post-infection, the S protein was detected in 60% of LC patients but not in controls. The amount of detectable S protein may be correlated with the number of organ systems implicated in symptoms (192). These findings suggest that viral persistence may be a key pathogenic mechanism that promote immune dysregulation observed in LC patients.

### 3.5 Role of mitochondrial dysfunction in LC

Studies have suggested a role of mitochondrial dysfunction as a possible explanation for the pathological manifestations in LC, which include fatigue and brain fog (193–195). One of the classical symptoms of LC is chronic fatigue, which can be linked to variation in mitochondrial ATP production (196), which inadvertently affects the proper functioning of the electron transport chain to promote the production of the reactive oxygen species (ROS) leading to oxidative stress. An increase in mitochondria-derived ROS can activate Nuclear Factor Kappa-light-chain-enhancer of activated B cells (NF-κB) and nucleotide-binding domain, leucine-rich–containing family, pyrin domain–containing-3 (NLRP3) inflammasome, which triggers downstream activation of cytokines and chronic inflammation (197). This disrupted function leading to increased levels of ROS can cause damage to cellular and molecular structures, including lipids, proteins, and DNA, contributing to the tissue damage commonly observed in LC patients (198–200).

Mitochondrial dysfunction has also been implicated in ME/CFS, a complex disorder characterized by fatigue, pain, and cognitive symptoms (14, 197, 201). Pathogens like EBV, Q fever, influenza and human beta-coronaviruses (e.g., SARS-CoV-1, MERS-CoV, and HCoV-OC43) have been associated with ME/CFS (202, 203).

Infectious mononucleosis, an acute viral illness most caused by the EBV and characterized by symptoms such as extreme fatigue, sore throat, fever and swollen lymph nodes, is strongly associated with ME/CFS; with approximately 13% of adolescents with acute EBV infection developing ME/CFS within six months (203). Evidence for mitochondrial damage in ME/CFS includes structural abnormalities in mitochondria (like swelling and disrupted cristae), reduced ATP production and impaired oxidative phosphorylation in muscle and immune cells [(like including CD8^+^ and CD4^+^ T cells, neutrophils, lymphoblasts, NK cells, and peripheral blood mononuclear cells (PBMCs)], and elevated levels of markers of oxidative stress and metabolic dysfunction (such as increased lactate and ROS) (204–206).

The overlap of symptoms between ME/CFS and LC suggests a shared aetiology driven by mitochondrial dysfunction. SARS-CoV-2 may disrupt mitochondrial dynamics by suppressing fission and inducing hyperfusion, which enhances oxidative phosphorylation to support viral replication but ultimately leads to bioenergetic failure and oxidative stress (197, 207). This mitochondrial dysfunction may create a conducive environment for reactivation of latent herpes viruses such as EBV and VZV, which are known to persist in immune cells like B cells and require intact mitochondrial function for latency maintenance (208, 209). When these latent viruses reactivate, they cause chronic immune activation through persistent viral antigen exposure and the production of cross-reactive autoantibodies. This ongoing immune response can result in inflammation and tissue damage, ultimately contributing to the persistent, multiorgan symptoms seen in both ME/CFS and LC (208, 209).

Fatigue, commonly reported by LC patients, closely mirrors that experienced by heart failure patients, where mitochondrial dysfunction is a well-established factor (210, 211). These findings suggest that mitochondrial dysfunction plays a key role in the persistent symptoms of LC.

### 3.6 Gut dysbiosis observed in LC

COVID-19 pandemic was also responsible for causing significant digestive issues, with patients reporting symptoms such as IBS, nausea, diarrhoea and vomiting. Researchers have associated this phenomenon with compromised gut microbiome leading to gut dysbiosis (212). Studies have shown significant alterations in the composition of gut microbiota in COVID-19 patients. Individuals experiencing LC have been found to have elevated amounts of *Ruminococcus gnavus* and *Bacteroides vulgatus*, along with reduced levels of *Faecalibacterium prausnitzii*, one of the most abundant and beneficial bacteria in the healthy gut, in contrast to pre-pandemic healthy controls; where gut dysbiosis is shown to persists for a minimum of 14 months (213). Moreover, a significant association exists between decreased amounts of butyrate, a short-chain fatty acid (SCFA) that supports the gut barrier and has anti-inflammatory effects, and prolonged symptoms of COVID-19 at the 6-month mark (212, 214).

ACE2 receptor commonly found in the lungs which acts as an entry receptor for SARS-CoV-2, is also expressed in the gut epithelial cells facilitating virus-induced damage within the gut microbiome (215). This could explain the gut microbiome disruptions seen months after acute infection (216). Viral RNA and protein were retrieved from the stool samples of patients months after the initial COVID-19 diagnosis (217), although speculation remains on its importance in LC pathogenesis. However, a study has shown the presence of the virus in the upper and lower intestine biopsies long after acute infection prompting the theory of the gut remaining a viral reservoir eliciting LC symptoms such as nausea, vomiting or IBS (187).

Though initially thought to only replicate in mammalian cells, recent research indicates that SARS-CoV-2 may also have the ability to infect and replicate in gut bacteria, much like a bacteriophage (218). This possible bacteriophagic activity might help create a lasting pool of the virus and could also be involved in the gut dysbiosis seen in people with LC. SARS-CoV-2 infection induces profound alterations in the gut microbiome, leading to dysbiosis characterized by reduced beneficial bacteria (such as *Faecalibacterium prausnitzii* and *Bifidobacterium* spp.) and increased opportunistic pathogens (such as *Enterococcus* and *Escherichia coli*). This disruption of microbial population is associated with heightened inflammation, impaired gut barrier function, and systemic immune activation, further exacerbating disease severity and post-acute SARS-CoV-2 infection, including endothelial dysfunction and hypercoagulation, which are implicated in LC (219).

## 4 Neurological implications of LC

Although the predominant presentation of SARS-CoV-2 is as a respiratory disease, it can involve an array of extrapulmonary organ dysfunction including neurological implications which can be classified as a part of LC profile (220–222). It is also important to note that as LC is considered as a multi-organ disease, neurological symptoms involving both the CNS and peripheral nervous system (PNS) could present alone or in conjunction with other organ systems including cardiorespiratory, renal, endocrine, dermatological, immunological or haematological systems (223–225).

The most common neurological symptoms include fatigue, headaches, tinnitus, vertigo, myalgia and disturbances in smell and taste which can occur during the acute phases of COVID-19 (226, 227). These symptoms vary in severity and can also interplay with other organ systems, but there is usually an improvement within the first 12 weeks (227, 228). Some of these symptoms also persist beyond the acute phase and could fall under neurological symptoms of LC, which include cognitive (including brain fog) and memory impairment, anosmia, hyposmia, confusion, fatigue, headaches, myalgia, autonomic dysfunction, and neuropsychiatric symptoms which include anxiety, depression, post-traumatic stress disorder (PTSD), and sleep disturbance (225, 227, 228). Furthermore, the risk of neurological diseases such as cerebrovascular accident (CVA) including stroke and transient ischaemic attack (TIA) is approximately 50% higher within the first 12 months after acute COVID-19 due to micro-emboli, blood-brain barrier (BBB) dysfunction and neuroinflammation (225, 227–232).

### 4.1 How SARS-CoV-2 interacts with the nervous system

**Figure 4:**
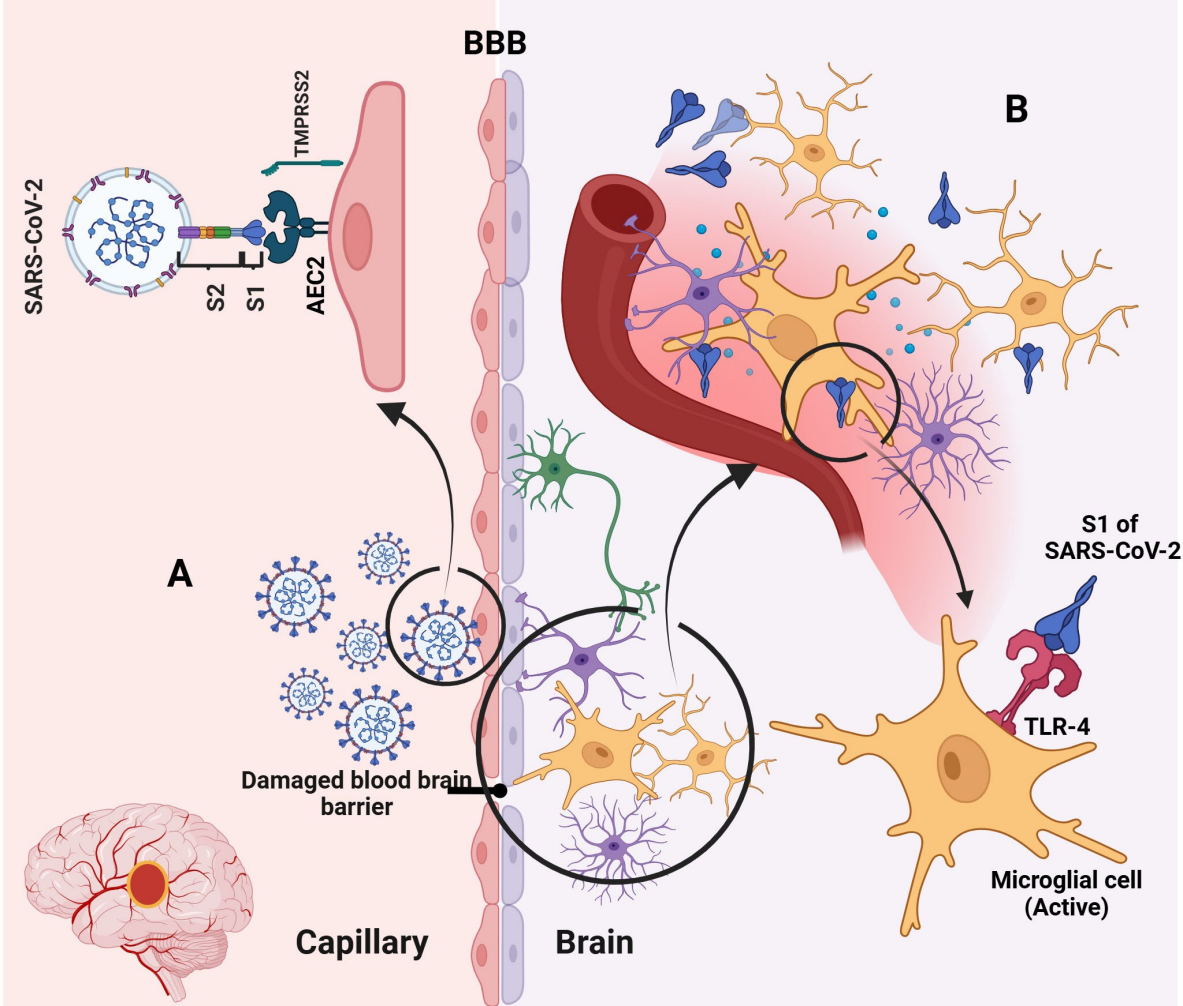
Potential pathophysiological changes which contribute to the neurological symptoms of acute and LC within the CNS. **(A)** During acute COVID-19, it is thought the SARS-CoV-2 virus can enter the central nervous system (CNS) via the hematogenous route or by engaging with angiotensin-converting enzyme 2 (ACE2) receptors on the surface of cells such as endothelial cells, smooth muscle cells, astrocytes, microglia, neurons and oligodendrocytes. ACE2 receptors are also expressed in regions such as the amygdala, brainstem, hippocampus, middle temporal gyrus, olfactory bulb and posterior cingulate cortex. **(B)** It is thought the pro-inflammatory state caused by the virus leads to blood brain barrier (BBB) dysfunction allowing infiltration of viral infected cells, cytokines and immune complexes. S 1 protein of the SARS-CoV-2 virus is thought to bind to Toll-like receptor 4 (TLR4) of microglia leading to microglial activation. Together with BBB dysfunction, microglial activation and a neuroinflammatory state, it is thought that this can cause potential neurodegeneration which could be contributing to the long-term neurological implications of LC. Created in https://BioRender.com.

**Figure 5:**
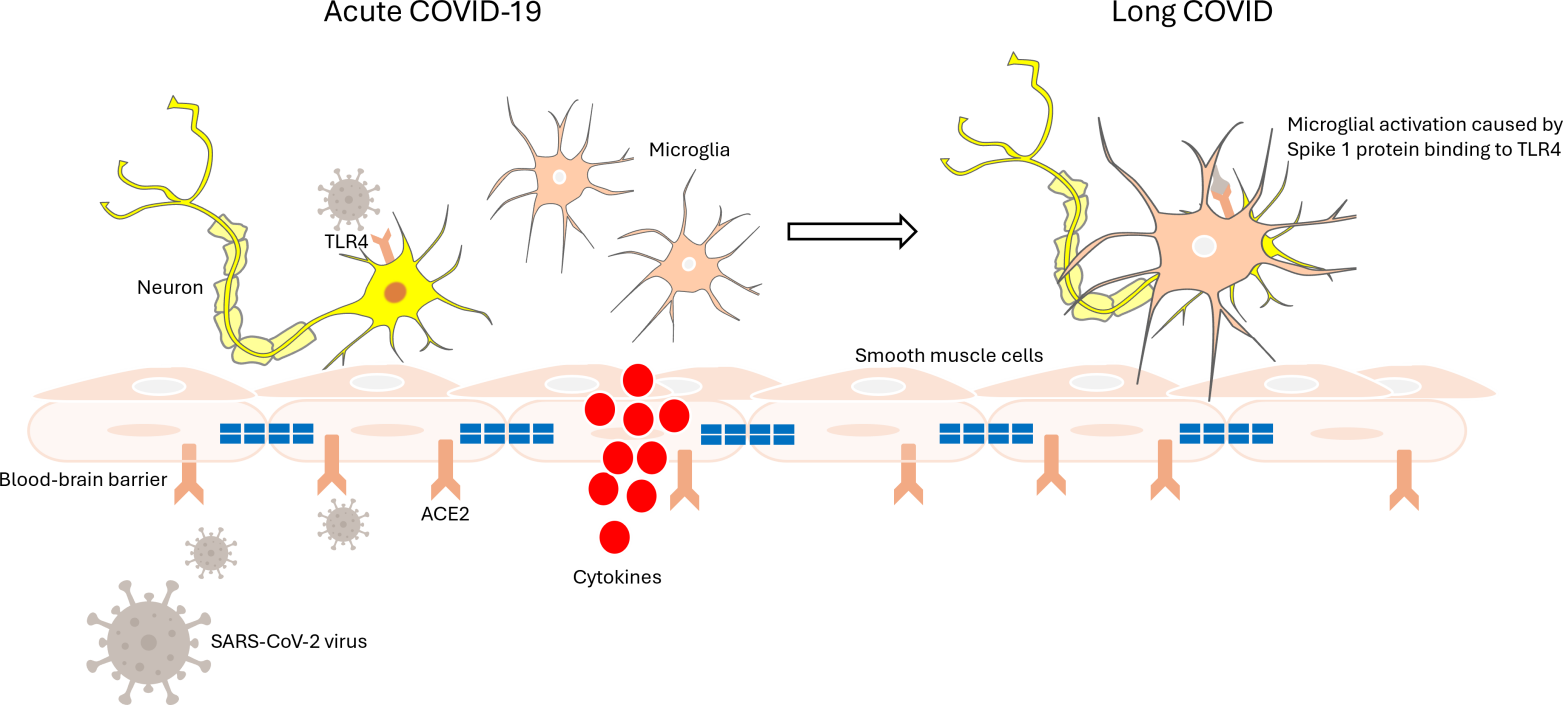
Potential pathophysiological changes which contribute to the neurological symptoms of acute and LC within the CNS. During acute COVID-19, it is thought the SARS-CoV-2 virus can enter the central nervous system (CNS) via the hematogenous route or by engaging with angiotensin-converting enzyme 2 (ACE2) receptors on the surface of cells such as endothelial cells, smooth muscle cells, astrocytes, microglia, neurons and oligodendrocytes. ACE2 receptors are also expressed in regions such as the amygdala, brainstem, hippocampus, middle temporal gyrus, olfactory bulb and posterior cingulate cortex. It is thought the pro-inflammatory state caused by the virus leads to blood brain barrier (BBB) dysfunction allowing infiltration of viral infected cells, cytokines and immune complexes. S 1 protein of the SARS-CoV-2 virus is thought to bind to Toll-like receptor 4 (TLR4) of microglia leading to microglial activation. Together with BBB dysfunction, microglial activation and a neuroinflammatory state, it is thought that this can cause potential neurodegeneration which could be contributing to the long-term neurological implications of LC.

SARS-CoV-2 virus can interact with human cells by engaging with the ACE2 receptors which are located on the surfaces of endothelial, smooth muscle, skeletal muscle, and CNS cells (**Figures 4**, **5**) (55, 233, 234). This engagement activates SARS-CoV-2 S protein by cleavage of TMPRSS2 as shown in **Figure 4** (55, 233, 234). Polymorphisms which alter ACE2 affinity towards SARS-CoV-2, such as K26R and T92I in the ACE2 gene, increases the receptor activity for SARS-CoV-2 S protein. Also, ACE2 polymorphisms such as rs2285666 that raises ACE2 expression, may enhance susceptibility to COVID-19. On the other hand, other variants like K31R, E37K and N33I could negatively affect S binding leading to lower disease susceptibility. TMPRSS2 variants such as rs12329760 and rs2106806 can also negatively impact S protein binding thereby inhibiting or slowing down viral entry. The genetic differences within ACE2 and TMPRSS2 can explain the population-level variability in susceptibility to and severity of COVID-19 (235).

Astrocytes, microglia, neurons and oligodendrocytes also have ACE2 receptors which may explain the neuro-invasion and neurovirulence of SARS-CoV-2 (55, 233, 234). This is further supported by animal model and cell culture studies (236). SARS-CoV-2 can invade the CNS through the haematogenous route due to BBB dysfunction via the choroid plexus and into cerebral circulation, and spread via the olfactory tract (236–238). BBB dysfunction can be caused by endotheliopathy due to pro-inflammatory responses to SARS-CoV-2, this subsequently allows infiltration of virus-infected cells, cytokines, as well as immune complexes from systemic circulation which activates the classical pathway of the complement system contributing to neuroinflammation (48, 236, 239). Within the CNS, ACE2 receptors are expressed in regions such as the amygdala, brainstem, hippocampus, middle temporal gyrus, olfactory bulb and posterior cingulate cortex; this could correlate with some of the neurological features in LC patients which include anosmia, hyposmia, autonomic dysfunction, cognitive and memory impairment, and neuropsychiatric symptoms (**Figure 5**) (240, 241). As mentioned above, anosmia and hyposmia are symptoms of LC. During the acute phase of COVID-19, SARS-CoV-2 virus can interact with cells in the olfactory epithelium which express ACE2; cells include sustentacular cells, stem cells and perivascular cells (242). This results in inflammation and subsequent olfactory epithelial damage, and thus, reduced olfactory bulb volume which could potentially explain anosmia and hyposmia in LC patients (243).

Neuroinflammation and neuroimmune response are thought to play a role in acute COVID-19 and LC via the interaction of neural and glial cells (229, 244). Microglia, the resident immune cells of the CNS, play a fundamental role in immune surveillance, apoptosis, neurogenesis, synaptic plasticity and synaptic pruning along with the complement system (245). Microglia have extended processes which they use to monitor the local environment for pathogens and cellular debris, and provide trophic support to the brain (245). Astrocytes are essential for CNS homeostasis as they play a role in neuronal development, provide metabolic support to synapses and contribute to the BBB via their astrocytic end-feet (245). The mechanisms by which SARS-CoV-2 invades the CNS and drives neuroinflammation are summarized in **Figure 4**. This depicts the pathways such as ACE2- and TLR4-mediated viral entry, BBB dysfunction, microglial activation, and the downstream neurodegenerative pathology as observed in LC.

Evidence suggests that SARS-CoV-2 contributes to dysregulation of the innate immune system via pro-inflammatory cytokines such as IL-1β and IL-6, which disrupt the BBB (246). IL-1β can act on neurons and astrocytes causing glutamate excitotoxicity and act on neural stem cells and reduce neurogenesis which is essential for memory and learning potentially contributing to some neurological implications of LC (245, 247). Microglia can perform immune surveillance via pattern recognition receptors (PRRs) which can detect pathogen-associated molecular patterns (PAMPs) leading to microglial response and pro-inflammatory cytokine release (245). It is thought the SARS-CoV-2 S protein subunit S1, may cross the BBB and be sensed by microglia via TLR4 causing microglial activation and neuroinflammation (248). In an animal model study using rats, they showed that the SARS-CoV-2 S1 protein alone can act as a PAMP and directly activate microglia and trigger inflammation in the brain. The neuroinflammatory response is associated with behavioural changes seen in both acute COVID-19 infection and LC, such as fatigue, brain fog, anxiety and social withdrawal. This study implicates the detrimental effects of the viral proteins circulating in the system which could explain the persistent neurological and psychological symptoms observed by continued microglial activation and inflammatory signalling, post-acute infection (249).

Another study reported that patients with neurological symptoms of LC such as brain fog, showed increased levels of serum C-C motif chemokine ligand 11 (CCL11). Administration of CCL11 in mice recreated LC neuropathology, such as hippocampal microglial activation, impaired neurogenesis, and oligodendrocyte loss, suggesting the role of the chemokine CCL11 in driving neural dysfunction (25).

Evidence of neuroinflammation such as astrogliosis and microglial nodules has been noted in brain studies of patients who died during the first wave of COVID-19 (250). Most patients had multifocal vascular damage which was indicated by the presence of serum proteins in the brain parenchyma, accompanied by widespread endothelial activation, platelet aggregation and microthrombi within the endothelial cell wall (250). Immunostaining identified complement components such as C1q in endothelial cells which was thought to be induced by SARS-CoV-2 S protein (250). C1q is involved in synaptic pruning and plasticity; the implications of complement system activation and neuroinflammation need to be examined further in the context of LC (245). Another study that examined novel brain organoid models with innately developing microglia exhibited microgliosis 72 hours after infection and threefold increase in microglial engulfment of postsynaptic termini and synapse destruction (251).

Cytokine abnormalities, endothelial dysfunction, elevated levels of CCL11 and ACE2 receptors in particular brain regions that correlate with neurological symptoms of LC are factors all of which can cause activation of glial cells and contribute to BBB disruption, potentially resulting in a chronic neuroinflammatory state which can be a vicious cycle leading to synaptic destruction, and potentially neurodegeneration over time.

### 4.2 Headache

A meta-analysis study which included 18,251 articles consisting of 47,910 individuals between the ages of 17 and 87 years concluded that headaches persisted in 44% post-COVID-19 infection (11). Another study identified that in a sample of 10,530 individuals with LC at the 12-week follow-up stage, approximately 15% still suffered from headaches (228, 229). It is thought that LC headaches could be a worsening of previous primary headaches, or potentially a new headache, which occurs daily or intermittently and starts during acute COVID-19 infection (252). These LC headaches are thought to present as tension-type headaches and could also have migraine-like features (252).

### 4.3 Anosmia and hyposmia

A reduction or loss of smell is thought to occur in approximately 12% of individuals in the aforementioned meta-analysis of 10,530 people affected by LC (228). It is thought that during acute infection, the virus causes a reduction or total loss of smell by altering gene expression in the primary olfactory neurons which are housed in the olfactory epithelium and responsible for detecting odours (253). A study that analysed olfactory tissue biopsies from individuals with persistent long-term loss of smell post-acute SARS-CoV-2 infection identified infiltration of CD8^+^ resident γδ T cells expressing IFN-γ, as well as a shift in the myeloid cell composition (253). The study also identified an increase in CD207^+^ dendritic cells and a decrease in anti-inflammatory M2 macrophages (253). However, there was no detectable SARS-CoV-2 RNA. This suggests that ongoing T-cell-mediated inflammation in the olfactory epithelium could be a cause of anosmia or hyposmia in LC patients (253).

### 4.4 Myalgia and fatigue

The meta-analysis study also identified that fatigue was the most common neurological symptom in LC individuals affecting 37% and myalgia affecting 17% (228). In a prospective cohort study of 81 patients one year on from acute SARS-CoV-2 infection, 38% still reported fatigue and 17% reported myalgia and muscle weakness (254). A meta-analysis study which comprised of 194 studies and approximately 725,000 individuals observed that fatigue was a symptom of LC in 38.4% of non-hospitalised individuals and 28.4% of hospitalised individuals at an average of 126 days follow-up (255). Fatigue has been linked with myopathy (256). In a small cohort study of 16 individuals, 50% of patients reported fatigue, myalgia or weakness for about 14 months post-COVID-19 infection and also had clinically detectable muscle weakness. Of those patients, approximately 85% of patients showed myopathic changes such as shortened myopathic motor unit potentials on electromyography (256). All the patients exhibited histopathological changes on muscle biopsies which included T-cell infiltration, leukocyte antigen ABC expression and atrophic and regenerating fibres (256). It is also important to note that fatigue is a non-specific complaint and quite subjective and could also encompass neuropsychiatric, cardiopulmonary and autonomic dysfunction (256).

### 4.5 Autonomic dysfunction

Symptoms of autonomic dysfunction such as tachycardia, gastrointestinal dysfunction, dizziness, abnormal sweating and flushing have been reported in patients with LC (257–259). A cross-sectional study reported a high prevalence of dysautonomia symptoms (76.7%), using the Composite Autonomic Symptom Score 31 questionnaire (COMPASS-31), where a score of >16.4 was suggestive that autonomic dysfunction may be associated with longer duration of LC (260). Another study that used the COMPASS-31 questionnaire on 2,314 individuals concluded that 66% of LC patients had a COMPASS-31 score of >20, suggesting moderate to severe autonomic dysfunction in LC patients (261).

### 4.6 Cognitive and memory impairment

Cognitive impairment which includes decline in executive function, working memory, attention and learning is a commonly reported symptom of LC (262–265). In one study, 194 patients were assessed on an average 7 months after acute COVID-19 infection; approximately 20-53% of the patients exhibited clinically relevant cognitive impairment which included executive functions and working memory and mild to moderate impairments in verbal fluency and learning, and memory when compared to healthy controls (265). Additionally, the study noted moderate to large impairment in global cognition, executive function and working memory (265). A recent study conducted by Vakani et al. (2025) investigated an association between persistent COVID-19 symptoms and brain structural volume by performing magnetic resonance imaging (MRI) on the brains of 43 working-age adults with a known history of COVID-19 and assessing cognitive functional domains (266). They concluded that higher persistent COVID-19 symptoms were significantly associated with smaller putamen volume and impaired cognitive functional domains (including executive function, memory performance and recognition) (266).

## 5 Development of LC depends upon time of vaccination

According to a recent study, the incidence of LC has decreased significantly over the period of the pandemic, declining from 10.42 per 100 individuals among the unvaccinated in the pre-Delta period to 3.50 per 100 individuals among the vaccinated in Omicron period. This decrease has been attributed to a widespread vaccination campaign globally (267).

The protective effect of COVID-19 vaccines against LC in individuals with mild to moderate infection has been confirmed. Vaccination prior to infection with SARS-CoV-2 can greatly reduce the risk of developing LC symptoms by 7–10 times even if only one dose of the vaccine was administered, compared to those who were unvaccinated (268). Administration of the vaccine 12 (268) to 20 weeks (269) prior to the infection has been linked with a significant reduction in chances of developing LC symptoms. Furthermore, patients who received two doses of the vaccine had better protection against LC in comparison to patients who received a single dose (270). The chances of having symptoms for more than 28 days in the vaccinated group were halved by two doses of the vaccines before the infection (271). An overall lower frequency and severity of symptoms has been reported in vaccinated patients who developed LC (272).

The effect of vaccination in patients who are currently affected affected by LC is not well understood. In one study, a reported 12.8% reduction in LC symptoms were observed following the first dose, which further reduced by 8% upon administration of second dose of the vaccine. Further, the second dose showed a weekly reduction of 0.8% in symptoms (273). On the other hand, worsening of LC symptoms on administration of vaccine has also been reported (274) which may be due to an excessive immune response.

Strain et al., 2022, explored how different types of COVID-19 vaccines affected patients reporting LC symptoms. Most participants reported improvements in LC symptoms after vaccination, and this effect was observed for all vaccine types. Patients who received the mRNA vaccine (Pfizer-BioNTech or Moderna) reported relief from LC-related symptoms such as fatigue, brain fog, myalgia, gastrointestinal symptoms, and autonomic dysfunction. Recipients of the AstraZeneca vaccine with LC reported an overall improvement in symptoms, but the average magnitude of symptom relief was less than that reported by recipients of mRNA vaccines. This study indicates that while all vaccines were primarily associated with protection, mRNA vaccines may offer a greater likelihood of symptom improvement for people living with LC, though individual responses still varied considerably (274).

A study of 42 patients with LC found that with a single COVID-19 vaccination, most patients experienced no symptom changes, while some reported improvement; however, around 21% reported worsening of LC symptoms such as fatigue and joint pain. Patients who reported worsening symptoms showed a significantly higher increase in SARS-CoV-2 S-specific antibody titres, suggesting a possible hyperactivated immune response to vaccination which could explain the symptom exacerbation. While most patients reported relief of LC symptoms, cases of worsening LC symptoms have also been reported, prompting the need for further research on the effect of vaccination on LC (275).

### 5.1 Booster doses and their effect on the LC syndrome

Recently, a study comprising of large-scale cohort data show that receiving a complete COVID-19 vaccination regimen especially with booster doses, leads to a progressively stronger immune response which is characterized by increased production of anti-spike antibodies and enhanced T-cell activity. Which significantly reduces the risk and persistence of long-term health consequences, including cardiovascular conditions and mortality, after SARS-CoV-2 infection compared to those who are unvaccinated or incompletely vaccinated (276). Booster doses are associated with a 26% reduction in the incidence of COVID-19 infection and a 75% reduction in severe cases of COVID-19 (277) which are very often linked with LC. Targeted administration of booster doses to high-risk groups like females, older adults, obese individuals and immunocompromised individuals who are more likely to develop LC symptoms (e.g., brain fog, fatigue, shortness of breath, pain, autonomic dysfunction, cognitive impairment, headache, blood clots, gastrointestinal issues, or persistent loss of smell or taste for at least three months after acute SARS-CoV-2 infection) has been proposed as a strategy to alleviate disease burden (278).

In another study, individuals who received a booster dose of Pfizer-BioNTech COVID-19 vaccine (BNT162b2) before a breakthrough infection showed faster recovery in comparison to those unvaccinated as well as a reduced likelihood of developing LC symptoms. Among individuals who had been vaccinated but had not recently received a booster, there was a trend toward improvement, but this did not reach a statistical significance, likely due to the time since their last vaccine dose (274).

Booster doses, thus may prove to be a promising strategy for reducing the risk and severity of LC. However, extensive research is required to understand their benefits and regulate administration.

## 6 Conclusions and perspectives

Despite a general perception that the threat of SARS-CoV-2 infection is over, a shadow continues to loom, representing a challenge for global healthcare system. LC affects multiple organ systems, with underlying mechanisms such as viral persistence, immune, and endothelial dysregulation leading in its pathophysiology. Much remains to be accomplished in stratifying and understanding LC, especially when its symptoms range from neurological (brain fog) to cardiovascular and respiratory complications. Since a sizeable proportion of COVID-19 survivors experience persistent LC symptoms, it is imperative to understand the underlying mechanisms and risk factors (279).

Researchers were able to identify several key pathophysiological mechanisms underlying SARS-CoV-2 infection such as the long-term effects of the involvement of SARS-CoV-2 virus with RAS through ACE2 receptor, which contributes to the development of cytokine storm syndrome and cellular damage. The association of ACE2 receptors with SARS-CoV-2 virus leads to an immune imbalance which favours the pro-inflammatory ACE/Ang II/AT1R axis, which partially explains the underlying long-term complications observed in COVID-19 patients, such as pulmonary inflammation, vasoconstriction, and multi-organ damage.

Latent EBV reactivation is another pathophysiological mechanism that has garnered some traction with respect to the persistent COVID-19-like symptoms. This ubiquitous herpesvirus establishes a lifelong latent reservoir in COVID-19 patients post-primary infection. Normally, these herpesvirus infections remain asymptomatic in its latent state, but EBV can reactivate under specific conditions of immune dysregulation or stress. The immune dysregulation generated as a part of the SARS-CoV-2 infection could potentially activate these latent viruses, further contributing to immune dysfunction (280). EBV could trigger a cascade of immune responses potentially aggravating the LC symptoms. Reactivated EBV can infect B and epithelial cells, leading to the production of pro-inflammatory cytokines, which could potentially lead to the development of a variety of symptoms of LC such as fatigue and cognitive dysfunction (281). Additionally, EBV reactivation has also been associated with the development of autoimmune disorders such as rheumatoid arthritis and Sjögren’s syndrome, which could further exacerbate the LC pathophysiology. Further research into the impact of latent herpesvirus, considering its reactivation potential which could significantly affect the immune system as described here could significantly contribute to our understanding of the diverse and persistent symptoms seen in LC.

While vaccination has been shown to significantly reduce the risk of acute SARS-CoV-2 infection, its impact on the incidence and severity of LC remains underexplored. Further research into how different vaccines and the number of doses received influence the likelihood of contracting LC is necessary for improving vaccination strategies and public health policies. In addition, the constantly evolving SARS-CoV-2 variants, its consequence on the development of LC and impact of vaccination status also need further investigation (7, 27, 28).

While the current understanding on the impact of LC on maternal and neonatal outcomes is limited, evidence has shown that maternal COVID-19 vaccination, particularly in the second and third trimesters, reduces disease escalation while eliciting humoral immune responses. In addition to the vaccine-induced IgG antibodies that are efficiently transferred across the placenta via FcRn-mediated transport, breast milk also contributes to mucosal IgA and IgG antibodies, which supports newborn development (282). Despite these benefits, acute SARS-CoV-2 infection remains associated with an increased risk of adverse pregnancy outcomes, although placental infection and vertical transmission are considerably rare (69, 283). However, questions remain on the extended effects and timing of vaccination, persistence of passive immunity on neonatal immunity and the impact of emerging variants.

Notably, all the persistent symptoms associated with LC need not exclusively be linked to LC. Patients who survived critical illness have been reported to develop post-intensive care syndrome (PICS) or post-sepsis syndrome (PSS), which are characterized by long-term physical, cognitive and psychological impairments (284, 285). Evidently, the symptoms observed in PICS and PSS overlap with LC symptomology, which also include fatigue, cognitive impairment and mental health disturbances (286). In addition, pathophysiological mechanisms underlying LC such as immune dysregulation, mitochondrial dysfunction and persistent inflammation are also observed symptoms of PICS (286). Lack of specific diagnostic biomarkers and the heterogeneity of symptoms involved in both LC, PICS and PSS make distinction between them difficult, and it is possible that the conditions coexist within the same patient (287). This overlap represents a critical limitation in the current understanding of LC and prompts the need for further research to distinguish between these conditions.

In conclusion, this review highlights the need for further research into LC to elucidate and clarify the underlying mechanisms, identify possible biomarkers, and develop better treatment options. Increased awareness of LC opens up a pathway that intertwines multiple pathophysiological mechanisms that contribute to its complex symptomology.

## Glossary

LC: Long COVID
PASC: Post-acute sequelae of COVID-19
ME/CFS: Myalgic Encephalomyelitis Chronic Fatigue Syndrome
WHO: World Health Organization
E: Envelope protein
M: Membrane protein
N: Nucleocapsid protein
S: Spike protein
RBD: Receptor-binding domain
ACE2: Angiotensin-converting enzyme 2
RAS: Renin angiotensin system
AT1R: Angiotensin II type 1 receptor
AT2R: Angiotensin II type 2 receptor
TMPRSS2: Transmembrane Serine Protease 2
Ang II: Angiotensin II
IL-1: Interleukin-1
IL-6: Interleukin-6
IL-12: Interleukin-12
IFN-γ: Interferon gamma
ARDS: Acute respiratory distress syndrome
SARS-CoV-2: Severe acute respiratory syndrome coronavirus 2
MERS: Middle East Respiratory Syndrome
TLR7: Toll-like receptor 7
REAP: Rapid Extracellular Antigen Profiling
PBMCs: Peripheral blood mononuclear cells
CMV: Cytomegalovirus
EBV: Epstein-Barr virus
VZV: Varicella Zoster Virus
NETs: Neutrophil extracellular traps
ANA: Anti-nuclear antibody
ACA: Anti-cytokine antibody
APLA: Anti-phospholipid antibody
RFs: Rheumatoid factors
GPCR: G protein-coupled receptor
POTS: Postural orthostatic tachycardia syndrome
PICS: Post-Intensive Care Syndrome
PSS: Post-Sepsis Syndrome
TCC: Terminal complement complex
NETosis: Neutrophil extracellular trap formation
RSV: Respiratory syncytial virus
HSV: Herpes simplex virus
HSV-1: Herpes simplex virus-1
PR-LE: Pityriasis rosea-like eruption
GD: Graves’ disease
IBS: Irritable bowel syndrome
ACPA: Anti-citrullinated protein antibody
tTG: Tissue transglutaminase
DGP: Deamidated gliadin peptide
TEMRA: Terminally differentiated effector memory T cells re-expressing CD45RA
cDC1: Conventional type 1 dendritic cells
PAMP: Pathogen-associated molecular pattern
APC: Antigen-presenting cell
TIGIT: T cell immunoreceptor with Ig and ITIM domains
TIM-3: T cell immunoglobulin and mucin domain-3
sTIM-3: Soluble T cell immunoglobulin and mucin domain-3
CTLA-4: Cytotoxic T-lymphocyte-associated protein 4
PD-1: Programmed cell death protein 1
LAG-3: Lymphocyte activation gene-3
TFH: T follicular helper cells
Treg: Regulatory T cells
cDC1: Conventional type 1 dendritic cells
TEMRA: Terminally differentiated effector memory T cells re-expressing CD45RA
MIS-C: Multisystem inflammatory syndrome in children
SLE: Systemic lupus erythematosus
RA: Rheumatoid arthritis
MS: Multiple sclerosis
SS: Sjögren’s syndrome
GD: Graves’ disease
IBD: Inflammatory bowel disease
CD: Celiac disease
T1D: Type 1 diabetes mellitus
PR-LE: Pityriasis rosea-like eruption
CXCR3: C-X-C motif chemokine receptor 3
CXCR4: C-X-C motif chemokine receptor 4
CXCR5: C-X-C motif chemokine receptor 5
CCR6: C-C motif chemokine receptor 6
TLR4: Toll-like receptor 4
PCR: Polymerase chain reaction
qPCR: Quantitative polymerase chain reaction
